# The Aim, Power and Instruments of Dental Educations, No. 1

**Published:** 1867-06

**Authors:** 


### AMERICAN JOURNAL

OF

### DENTAL SCIENCE.

Vol. I. THIRD SERIES.—JUNE, 1867. No. 2.

### ORIGINAL COMMUNICATIONS.

### ARTICLE I.

### *The Aim, Power and Instruments of Dental Education, No.* 1.

By Professor AUSTEN.

I PROPOSE to consider in these papers a subject to which every thoughtful dentist, who loves his profession, must give a very earnest consideration. None can put it from them by saying that it is no concern of theirs, but a matter for magazines and colleges. Every dentist, the most illiterate equally with the finest scholar; the most social and communicative not more than the reserved and exclusive—all are alike raised or lowered by whatever promotes or retards professional improvement; and among the agencies operating thereon, none are comparable in power with education.

In assuming for EDUCATION the first place among such agencies, I must be understood as using the word in its highest sense. *Instruction* may be considered according to either of the several modifications of the original signification of the Latin word: 1—to set in order, or arrange; 2—to construct, or build; 3—to prepare, furnish or equip.

In each of these primary meanings, we find elements of that secondary signification, which in the English language is its only one. For it is the duty of the instructor to "set in order and arrange" the knowledge which he imparts; "to construct or build" according to correct rules, the system, science or art which he teaches; "to prepare, furnish or equip" the mind and hand of his pupil.

Instruction, however, is not all of education. It defines the duty of the teacher; but in the word *education*, we find implied the corresponding duty of the student. This word is also of Latin origin, and to learn its true significance we must refer to its primary meaning. When Cicero says "educate (draw out) the sword from the scabbard," and Virgil says "educate (draw out) the weapon from the body," the literal translation sounds strangely, because in English the secondary meaning has supplanted the primary one. Yet when we say "educate (draw out) the mind," we find the meaning of Cicero and Virgil underlaying and giving force to the English word. The most complete instruction avails as little as the sword in its scabbard, unless the student will unite with the teacher in acquiring the ability to draw and wield it.

Education therefore differs essentially from instruction in this—whereas instruction does not necessarily require more from the student than diligence, attention and memory, his education is impossible, unless he will earnestly co-operate with his preceptor in the effort to "draw out," cultivate, quicken, enlarge, strengthen, develope and improve his mental and physical powers. Education gives the power to use and apply instruction.

It is clear that education is a Partnership, in which either party has the power to aid or to hinder, or even to neutralize the work of the other. Hence all schemes of education, and all theories as to its effect upon any given profession will fail, unless we have regard not only to the methods of teaching, but also to the character of those taught. So far from questioning the importance of correct instruction, it will be the special object of these papers to inquire into the best means of perfecting the same. But it should be distinctly understood and emphatically stated that, whilst laborious instruction "may put into" a dull mind much knowledge, no amount of education can "draw out of" him what does not exist.

If then, a profession looks to any educational system for its elevation, it must be remembered that quite as much depends upon the material furnished as upon the manner of its treatment, The training of asses cannot shorten their ears nor make musical their discordant bray; neither will many series of generations, by any law of transmutation of species, convert them into war horses. In justice to our methods of instruction, the imperfections of which are freely admitted, we must state plainly what is the duty of the dental profession in this important respect of selecting the material out of which the next professional generation is to be formed.

In every profession, exceptional men stand above it. They do it honour and help to elevate it, but cannot possibly raise it to their level. Again, exceptional men stand below and help to degrade. But the profession itself takes the level of its majority. Socially, it is refined or vulgar; intellectually, it is cultivated or ignorant; professionally, it is experienced or incompetent, according to the character of the majority. Hence we find an infinite variety in the local estimation of a given profession, dependent upon its average character in that locality. I have spoken of position, social intellectual and professional. Much foolish talk on the subject of the status of the dental profession arises from confounding these distinctions, owing in great part to the failure to distinguish between professional and personal character.

The distinctions and exclusions of social caste are not proper subjects of professional inquiry, and form no part of professional ambition. We should not consent to take our standing according to rules which can permit an abandoned roué to take precedence of Michael Faraday, or a corrupt politician to be more sought after than Sir Benjamin Brodie. Still, an honorable position in society is desired by all, and this every man may make for himself by virtue of his *individual* character. There are social circles which rigidly exclude certain professions; but this ban will not prevent any one from enjoying the high esteem of his fellow-men, if he shall choose to deserve it.

The honours awarded to intellect are more justly and less capriciously given. They are within the reach of every professional man who will work for them. It is worse than folly for any set of men to complain that the world does not rate them equal intellectually to some other set. It is the meanest kind of mental agrarianism; the idle and poor, clamorous for that wealth of reputation, which has been earned by genius and cultivated talent. When most surgeons were barbers, there was here and there an Ambrose Parè, but as a class they were not educated men. In spite of Celsus, Galen, Sydenham and Harvey, the medical profession in olden times took the intellectual and social rank of its majority, and this was by no means high. Two centuries of high mental culture and profound study of professional and collateral science filled the ranks of medicine and surgery with names that compelled the admiration of the world. The profession then took the rank of its majority, and the name of physician or surgeon, so far from a reproach, became a presumption of merit.

But how is it now? This hard-earned position has to struggle under a fearful weight of mediocrity and worse than mediocrity. And this because of the wide-spread perversion of the purpose and methods of medical education: because the refuse of our youth are permitted to pass through medical schools: because a young man who is too bad for the pulpit, too dull for the bar, too lazy for the store, and too ignorant for any other business or profession is thought quite equal to the *study* of medicine; and because so many medical schools pronounce him quite equal also to the *practice* of it. A few more years of persistence in this course will make men ashamed of the title of Doctor, and compel earnest workers to claim some other title.

The history of medicine thus proves that the position of every profession depends upon the proper selection of its material quite as much as, if not more than, upon the preparation of that material: and this because of the fact, that success in teaching depends so largely upon the co-operation of those taught. The province of professional teaching is the developement of *professional* capacity: this is as much as the term of study will permit. But the individual character is inseparable from the professional, and so constantly modifies it, that a certain degree of education thereof is indispensible as a foundation upon which to build.

The profession that would not retrogade must therefore make careful selection of the material which it proposes to educate for its specific duties. Hence if Dentistry wishes to be truly progressive it must not admit to its offices and colleges as indiscriminately as Medicine has in this country for thirty years done: and it should not pronounce EDUCATED those who really have no abilities that education can "draw out." How is this to be accomplished? Mainly by the individual and combined action of the members of the profession in the rejection of students, who have not the proper basis upon which to build a professional course of training.

I state the question independently of collegiate training, which will be considered hereafter, and assert that unless dentists UNITE in requiring of all students more *preparatory* qualification, than they now do, no amount of instruction whether of office or college will avail much towards the elevation of the standard of professional excellence. Until we make the meshes of our drag-net larger, we shall be overrun with "small fry;" for no amount of cramming can make a shad out of a gudgeon.

Since writing the above paragraph I have read this morning, (May 9,) that the Convention of Medical Teachers assembled in Cincinnati, have proposed resolutions acknowledging the evil above-named, and looking to its correction. The Convention of Dental Colleges did the same at its session in March last. The American Dental Association, through the circular of its committee, issued January 10th of this year, made an admirable appeal to the profession in the same direction.

Now there are doubtless dentists not yet prepared to respond to that appeal of the Association. There are medical schools that will not feel themelves bound by the resolutions of the Cincinnati Convention: just as there was one dental school that "seceded from the Union" of dental colleges. But the movement, first begun by the dental profession, and now vigorously urged by the medical, will steadily advance towards the true aim and end of all professional education—THE SELECTION AND PREPARATION OF THE MATERIAL OUT OF WHICH THE PROFESSION IS MADE.

For a while, perhaps permanently, offices will have fewer pupils and colleges fewer students, and both students and professors will find their work more laborious. But teachers, who love their work, will not complain, and students have a "margin" of spare time, which the devil often uses to their harm. As to the diminution of the number of students, this would be a blessing to medicine, and could do no harm to dentistry. There can be no more fatal error than to judge the merits of a school by the size of its classes: and no more pernicious practice, than to adopt regulations, which shall invite the largest number of matriculants, and secure the greatest proportion of graduates.

An office certificate or a college diploma is valuable in proportion to the difficulty of getting it; for no one prizes what anybody may have. A medical diploma has become worthless as a guarantee of fitness, and a dental diploma has not the value it should have. To have studied in the office of the best dentist gives no assurance of proficiency, so long as we know how little time the engrossing professional duties of such men permit them to give to students. The whole scheme of professional education, has long been defective and retrograding; but the tide has now turned, and we may from this date hope to witness a steady and rapid improvement.

This improvement may be expected to consist—

1. In a more careful selection and preparatory training of students.

2. In a more thorough education of students.

3. In a more careful inquiry into the results of that education; in other words, in more rigid examinations.

The first point has been the subject of this paper. It remains to consider the other two; after which I propose to inquire into the true significance, value and benefit of Diplomas, whether conferred by schools, colleges or associations.

